# Reliability of low‐power cycling efficiency in energy expenditure phenotyping of inactive men and women

**DOI:** 10.14814/phy2.13233

**Published:** 2017-05-14

**Authors:** Elie‐Jacques Fares, Laurie Isacco, Cathriona R. Monnard, Jennifer L. Miles‐Chan, Jean‐Pierre Montani, Yves Schutz, Abdul G. Dulloo

**Affiliations:** ^1^ Department of Medicine/PhysiologyUniversity of FribourgFribourgSwitzerland; ^2^ EA3920 and EPSI platformBourgogne Franche‐Comté UniversityBesançonFrance

**Keywords:** Energy expenditure, obesity, physical activity, sedentary, thermogenesis

## Abstract

Standardized approaches to assess human energy expenditure (EE) are well defined at rest and at moderate to high‐intensity exercise, but not at light intensity physical activities energetically comparable with those of daily life (i.e., 1.5–4 times the resting EE, i.e., 1.5–4 METs). Our aim was to validate a graded exercise test for assessing the energy cost of low‐intensity dynamic work in physically inactive humans, that is, those who habitually do not meet the guidelines for moderate‐to‐vigorous aerobic physical activity levels. In healthy and inactive young men and women (*n* = 55; aged 18–32 years), EE was assessed in the overnight‐fasted state by indirect calorimetry at rest and during graded cycling between 5 and 50W for 5 min at each power output on a bicycle ergometer. Repeatability was investigated on three separate days, and the effect of cadence was investigated in the range of 40–90 rpm. Within the low power range of cycling, all subjects perceived the exercise test as “light” on the Borg scale, the preferred cadence being 60 rpm. A strong linearity of the EE‐power relationship was observed between 10 and 50 W for each individual (*r* > 0.98), and the calculation of delta efficiency (DE) from the regression slope indicated that DE was similar in men and women (~29%). DE showed modest inter‐individual variability with a coefficient of variation (CV) of 11%, and a low intra‐individual variability with a CV of ~ 5%. No habituation or learning effect was observed in DE across days. In conclusion, the assessment of the efficiency of low power cycling by linear regression – and conducted within the range of EE observed for low‐intensity movements of everyday life (1.5–4 METs) – extends the capacity for metabolic phenotyping in the inactive population.

## Introduction

Variability in the efficiency of energy metabolism is often implicated in human susceptibility to leanness and fatness (Stock 1999; Blundell and Cooling 2000; Dulloo et al. 2012). Such variability in metabolic efficiency can be investigated by measuring energy expenditure (EE) under standardized conditions at rest, whether in the pre‐prandial (post‐absorptive) state as basal metabolic rate (Henry 2005) or in the post‐prandial state as thermic effect of food(de Jonge and Bray 1997; Schutz and Dulloo 2014). Its assessment in the non‐resting state, however, remains ill‐defined.

Indeed, the assessment of exercise efficiency during cyclic movement is performed using different exercise modalities such as treadmill walking (Donovan and Brooks 1977; Chen et al. 2004), running (Margaria et al. 1963; Cavagna et al. 1976) or cycling ergometry (Gaesser and Brooks 1975; Coyle et al. 1992; Moseley et al. 2004) with some studies reporting exercise efficiency as gross efficiency and others as net or delta efficiency (Suzuki 1979; Poole and Henson 1988; Nickleberry and Brooks 1996; Goldsmith et al. 2010). Furthermore, these exercise tests are most often performed at intensities well above those that correspond to non‐resting EE for most people living in modern societies, where their low‐level physical activities rarely exceed four times the resting metabolic rate value, that is, 4 METs, (Levine et al. 2000, 2001; Goh et al. 2016); these include fidgeting, postural transitions, office‐related or domestic house‐hold activities (e.g., cleaning, washing, cooking) and occupational‐related or leisure‐related walking at low or moderate speed (Levine et al. 2000, 2001; Goh et al. 2016).

However, such low‐intensity physical activities ‐ collectively referred to as Non‐exercise activity thermogenesis ‐ are increasingly recognized as playing an important role in weight regulation (Villablanca et al. 2015), and that their substitution by more sedentary behavior (mostly sitting or lying awake) seems to exert a greater influence on the epidemic of obesity and cardiometabolic diseases than moderate‐to‐vigorous intensity leisure‐time pursuits (Hamilton et al. 2007; Stamatakis et al. 2009; Wilmot et al. 2012; Henson et al. 2016). These findings have generated considerable interest for better monitoring, characterizing and promoting low‐level physical activities in daily life (Levine et al. 2000, 2001; Tudor‐Locke et al. 2014; Villablanca et al. 2015). Little attention, however, has been directed at assessing how humans vary in the specific energy cost (or efficiency) of performing low‐level physical activities, which, in addition to variations in intensity and duration of the physical activity, can also contribute to human predisposition to obesity.

There is therefore a need to develop approaches for EE phenotyping in response to standardized exercise at low power outputs, namely in the range of 1.5‐4 METs, to evaluate the reliability, repeatability and acceptability of such a test for untrained sedentary individuals, and thus to extend the capacity for EE phenotyping in the non‐resting state, energetically comparable to everyday life physical activities.

In this context, leg cycling in the range of low power output is a non‐weight‐bearing movement that can easily be performed by most people (trained or untrained) and well tolerated (Thivel et al. 2016). It is easier to standardize than other modes of physical activity and provides an accurate measurement of the external work performed. Furthermore, delta efficiency (DE) – which is calculated from the slope of this EE‐power linear regression (Gaesser and Brooks 1975) ‐ is often considered as the best indicator of muscle efficiency since the slope reflects the energy cost of biological processes that increase as power output increases (Gaesser and Brooks 1975; Coyle et al. 1992; Castronovo et al. 2013).

The objective of the study reported here was to assess the reliability of DE during low‐power cycling as a potential standardized approach for EE phenotyping in the range of daily EE typical of the general (sedentary) population. We first validated, specifically in inactive young men and women, the linearity of the EE‐power relationship in the range of low power output cycling, and the impact of cadence on this relationship. We subsequently investigated the extent to which DE varies in the same individual (i.e., its repeatability). Furthermore, we explored the extent to which any important inter‐individual variability in DE during this cycling exercise may be influenced by anthropometry and body composition.

## Methods

### Subjects

The experiments were conducted in a total of 55 healthy young adults (26 men and 29 women), aged 18–32y (Mean ± SEM: 24.2 ± 0.4), and BMI between 16.4 and 30.8 kg/m^2^ (Mean ± SEM: 22.1 ± 0.4); in women, the exercise tests were performed during the follicular phase of the menstrual cycle. The subjects were nonsmokers, had no previous history of cardiovascular events or any limitation on physical ability, were not taking supplements or medicine that might affect their metabolic rate, had stable body weight (defined as <3% variation during the past 6 months), were not pregnant nor breastfeeding. The selection of subjects as “inactive” was done through interview and the completion of a diet and lifestyle questionnaire that included habitual physical activity, with specific focus on time spent on moderate‐to‐vigorous aerobic physical activity (MVPA). The subjects were considered to be “inactive” in accordance to the proposal of the Sedentary Behaviour Research Network (2012) in referring to individuals who habitually do not perform sufficient amounts of MVPA, and who in our study did not meet the Canadian Physical Activity Guidelines (Tremblay et al. 2011), namely a maximum of 150 min of MVPA per week, in bouts of 10 min or more. All procedures were followed in accordance with the Helsinki Declaration and were approved by the state ethic committee (protocol 214/14); informed consent was obtained from all participants.

### Anthropometry and body composition

In the week preceding the first test day, the participants visited the laboratory to complete a questionnaire regarding their diet/lifestyle and medical history, and to familiarize themselves with the experimental procedures and equipment. After voiding the bladder, body weight and height were measured using a mechanical column scale with integrated stadiometer (Seca model 709, Hamburg, Germany), body composition using a multi‐frequency bioelectrical impedance analysis (Inbody 720, Biospace Co., Ltd, Seoul, Korea), and Trunk (abdominal) fat percentage by bioelectrical impedance analysis using ViScan (Tanita Corporation, Tokya, Japan).

### General study design

All participants were requested to avoid any strenuous physical activity, caffeine and dietary supplements in the 24 h before testing. Furthermore, to minimize the effect of physical activity on the morning of each test day, participants were requested to use motorized transport instead of walking or cycling to reach the laboratory. On the day of testing, subjects came to the laboratory in the morning (between 08:00 and 08:15) after a 10–12 h overnight fast. After at least 30 min rest, oxygen consumption and carbon dioxide were then measured by indirect calorimeter (Quark CPET Cosmed, Rome, Italy) using a Hans Rudolph silicon facemask for 10 min while seated at rest on a bicycle ergometer (Cosmed E100 P) and during the subsequent graded cycling exercise; EE was calculated according to the Weir equation (Weir 1949). Values of EE were averaged over the last 5 min of the resting period (no cycling) and over the last 2 min of cycling at each power output; the average EE (kcal/min) values were then plotted against power (W).

### Calculations of various expressions of efficiency

From the EE data (as kcal/min) for each subject, and conversion of mechanical power from watts to kcal/min, the various expressions of exercise efficiency were calculated. Gross, net, and work efficiency were calculated as defined by Gaesser and Brooks (1975), while delta efficiency was determined using two approaches, namely (1) the delta efficiency (referred to as DE) was obtained from the reciprocal of the slope of linear regression between EE (*y*‐axis) and mechanical power (*x*‐axis) for trials involving 10–50 W or 10–40 W, with 5 or 10 W increments, and efficiency values expressed as a percentage (Gaesser and Brooks 1975), and (2) the classical delta efficiency (referred to as DE‐md; i.e., by method of difference between two measurement trials) was obtained as the difference in mechanical power output from one trial to the next, divided by the associated change in EE over these two trials, and expressed as a percentage (Gaesser and Brooks 1975).

### Experiment I: Linearity of EE‐power & effort perception

Fifteen subjects (seven men and eight women) performed graded cycling at 60 revolutions per min (rpm) for 5 min at no‐load (theoretically 0 W) followed by 5, 10, 15, 20, 30, 40 and 50 W, respectively. In addition to EE, heart rate (HR) was measured throughout the protocol by a wireless physiological monitoring system (Equivital EQ‐01, Hidalgo, Cambridgeshire, UK). At the end of each power output, measures of perceived exertion were taken using the Borg scale (Borg 1982).

### Experiment II: Effect of cadence

This experiment was performed as two separate and sequential protocols. In the first protocol (Protocol I), 12 subjects (six men, six women) pedaled at increasing cadence (40, 60, 80 rpm) at either 10, 20, 30 or 40W for 5 min each in an increasing work rate sequence. In the second protocol (Protocol II), 10 subjects (seven men, three women) pedaled sequentially at 60, 90, and 60 rpm at either 10, 20, 30 or 40 W for 5 min each. Cadence preference was assessed verbally at the end of each test. Six subjects participated in both protocols.

### Experiment III: repeatability & habituation

Six subjects (three men, three women) were studied on three separate days, with at least 2 days interval, for the repeatability and habituation validation test. On each day, they pedaled at 60 rpm for 5 min each at 10, 20, 30, and 40 W. On Day 3, in addition to performing the increasing power output cycling phase (ascending) as on Days 1 and 2, subjects also performed a decreasing power output (descending) phase (40, 30, 20, 10 W), with these two phases separated by 30 min of rest in a comfortable chair.

### Experiment IV: Potential determinants of Inter‐individual variability in DE

The potential anthropometric and body composition determinants of inter‐individual variability DE, assessed by linear regression of EE versus power during graded cycling exercise in the low power range, namely at 10, 20, 30, and 40 W, was investigated in a total of 55 subjects (26 men and 29 women), which included the subjects recruited in the above experiments.

### Data and statistical analysis

The data are presented as Mean ± SEM, except where indicated. Statistical analyses were performed using the computer software STATISTIX version 8.0 (Analytical Software, St Paul, Minnesota, USA). Analysis of EE against power was performed by ANOVA with repeated measures (with or without cadence as a within‐subject factor, and with or without gender as a between‐subject factor). All reported *P* values are two‐sided. For all tests, significance was set at *P* ≤ 0.05.

## Results

### Experiment I: linearity of EE‐power & perception

**Figure 1:**
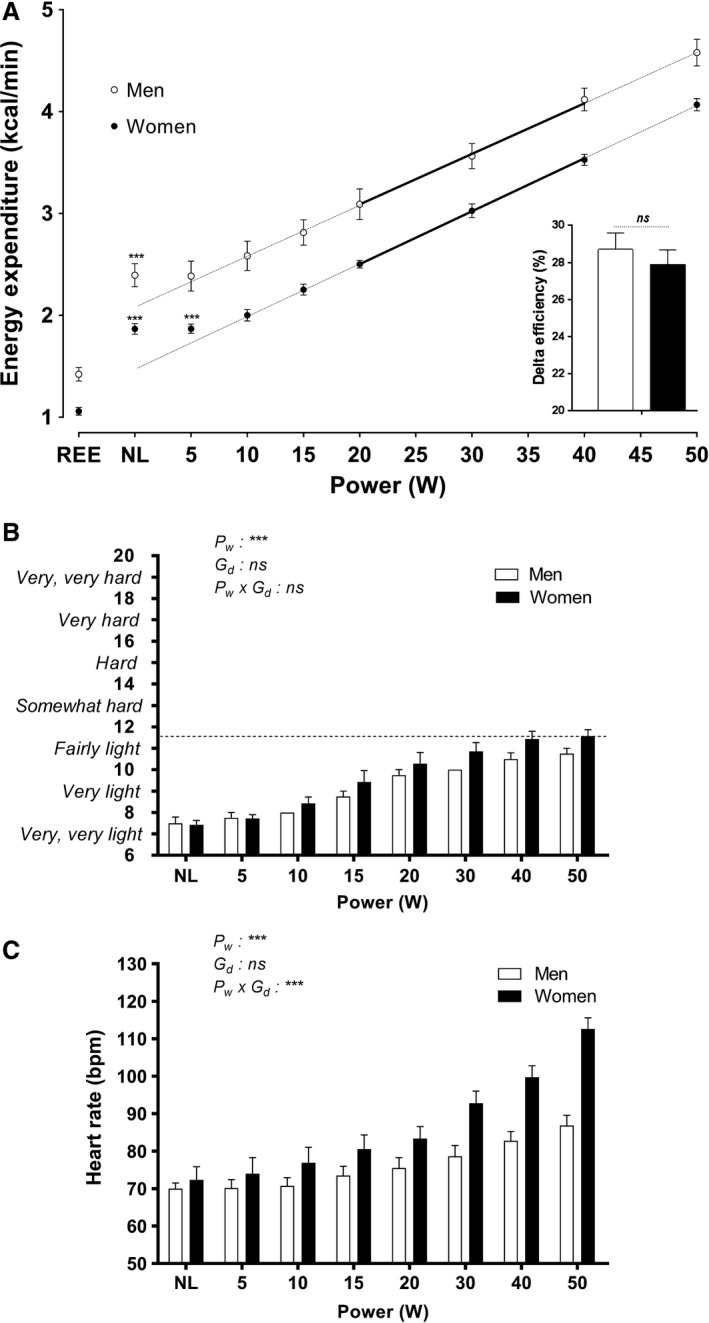
*Panel* A: Effect of power on energy expenditure and delta efficiency (DE) in 15 participants; seven men (white circles/bars), eight women (black circles/bars). Values are Mean ± SEM. REE=resting energy expenditure; NL=No‐Load. The equations of the linear regression (20–40 W) are as follows: Men: Y = 0.05139*X + 2.049, R^2^ = 0.9979; and women: Y = 0.05123*X + 1.482, *R* ^2^ = 0.9998. Predicted and measured values were significantly different in both men and women at NL and in women at 5W. Delta Efficiency values were similar in both genders during 10–50W power output interval. Borg Scale of Perceived Exertion values *(Panel B)* and heart rate *(Panel C)* at different power outputs in men (white bars), and women (black bars).Values are Mean ± SEM. For Perceived Exertion, repeated measures ANOVA indicates a significant effect of power (P_w_), with no significant gender effect (G_d_) or interaction (P_w_ x G_d_). For heart rate, repeated measures ANOVA indicates significant effect of P_w_ as well as P_w_ x G_d_ interaction effect.

**Figure 2:**
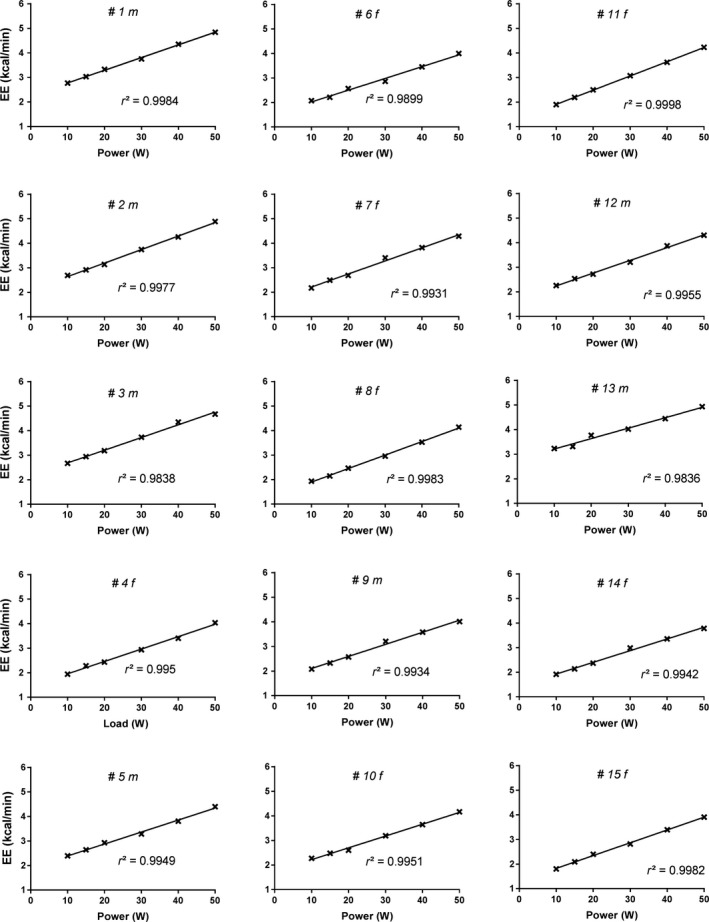
Individual linear regressions of energy expenditure (EE) versus power output for 15 subjects; the “m” and “f” after subject number denoting male and female, respectively.

In our investigation of the linearity of the EE‐power relationship in the low power range of 5–50 W (Fig.**** 1, panel A), we performed data analysis based upon the findings of a preliminary experiment indicating that EE values during 20, 30 and 40 W were almost perfectly linearly aligned. Consequently, we first regressed our EE data in this 20–40 W range, and show here that these EE data at 20, 30 and 40 W were also almost perfectly linearly aligned both in men and women. We then tested whether or not the measured values for EE at lower power outputs (5, 10, 15 W) or at higher power output (50 W) were different from the EE values predicted from this linear EE‐power relationship (20–40 W). A significant difference between measured and predicted EE was observed at 5 W in women only. Furthermore, in both men and women, the no‐load (NL) values were higher than those predicted for 0 W. Thus, a very strong linearity of the EE versus power output relationship was observed between 10 and 50 W in both men and women; with EE relative to measured resting EE (REE) corresponding to 1.8 and 3.8 METs at 10 and 50 W, respectively. For each of the 15 individuals, the r^2^ value of this EE‐power linear relationship ‐ based upon five data points ‐ exceeded 0.98 (Fig.**** 2). While there was, as expected, a significant gender difference in y‐intercept (*P* < 0.01), there was no gender difference in the slope and hence in DE which, on average, was about 28.4%.

Within the range of cycling considered as low power (10‐50W), EE relative to resting EE corresponding to 2‐4 METs and the perceived exertion, assessed on the Borg scale, ranged from “very, very light” to “fairly light” in both men and women, as shown in Figure 1 (panel B). Perceived exertion ratings were slightly higher in women than men, and although this gender difference did not reach statistical significance, it was nonetheless reflected in heart rate values (Fig. 1, panel C), which were significantly higher in women compared to men particularly in the upper range of power (ANOVA power x gender interaction: *P* < 0.001). However, even at the highest level of exercise intensity in this low power range (50 W), perceived exertion remained below the score of 12 on the Borg scale, close to or below “fairly light” in both genders. Overall, within the low power output range of cycling (5–50 W), which was perceived by all subject to be “light”, a very strong linearity of the EE versus power output relationship was observed between 10 and 50 W in both men and women.

**Figure 3:**
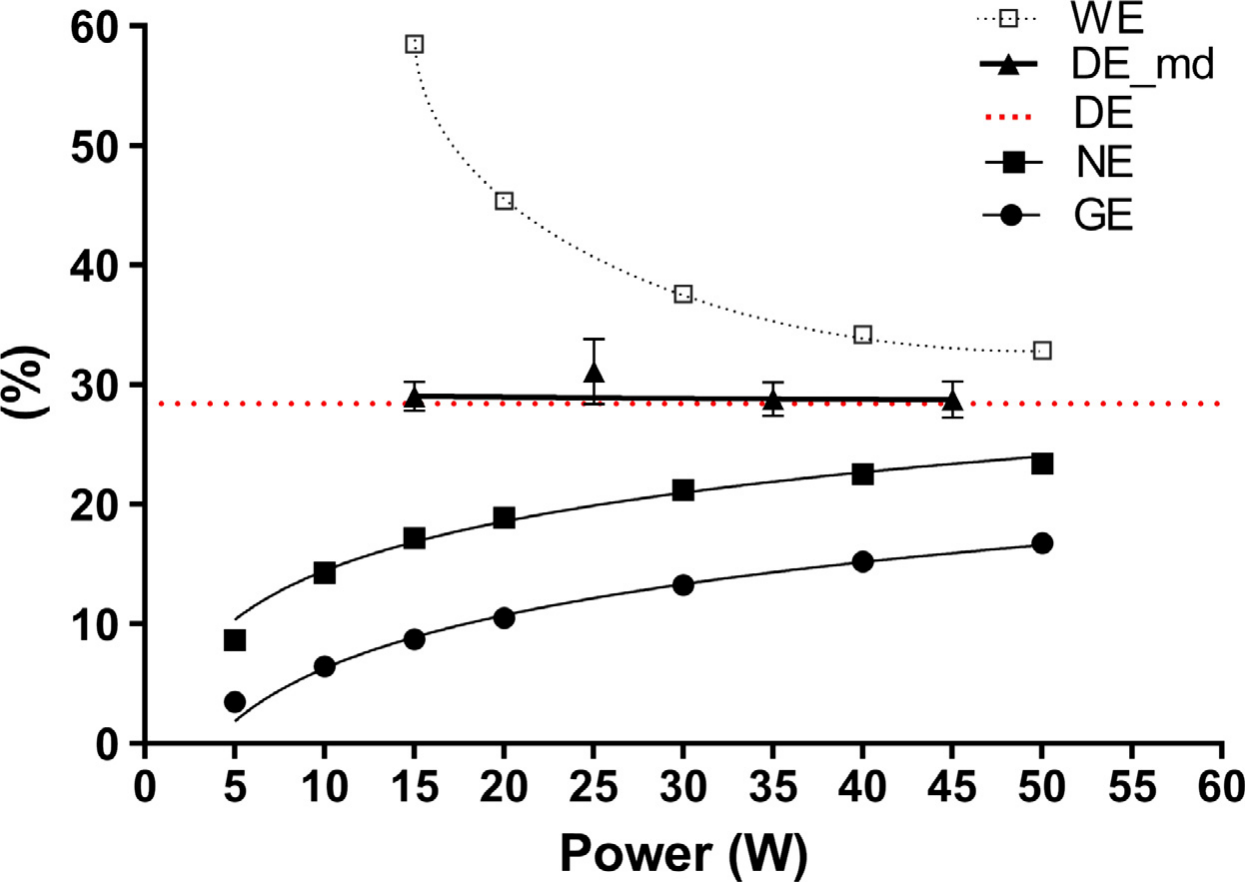
Exercise efficiency values versus power output. WE= work efficiency, DE‐md= delta efficiency calculated by the method of difference, DE= delta efficiency assessed by linear regression (dotted red line), NE, net efficiency, GE, gross efficiency.

The values of cycling exercise efficiency across this low power range, expressed in other ways are shown for all subjects in Figure**** 3; the value for DE (by regression) being represented by the dotted horizontal line. The values for both gross efficiency (GE) and net efficiency (NE) increased in a curvilinear manner with increasing power. The values for delta efficiency calculated by method of difference across two consecutive power loads (DE‐md) was found to be relatively constant. However, values of work efficiency (WE) were found to be much higher at the lowest power outputs (<30 W) and decreased in a curvilinear manner.

### Experiment II: effect of cadence

**Figure 4:**
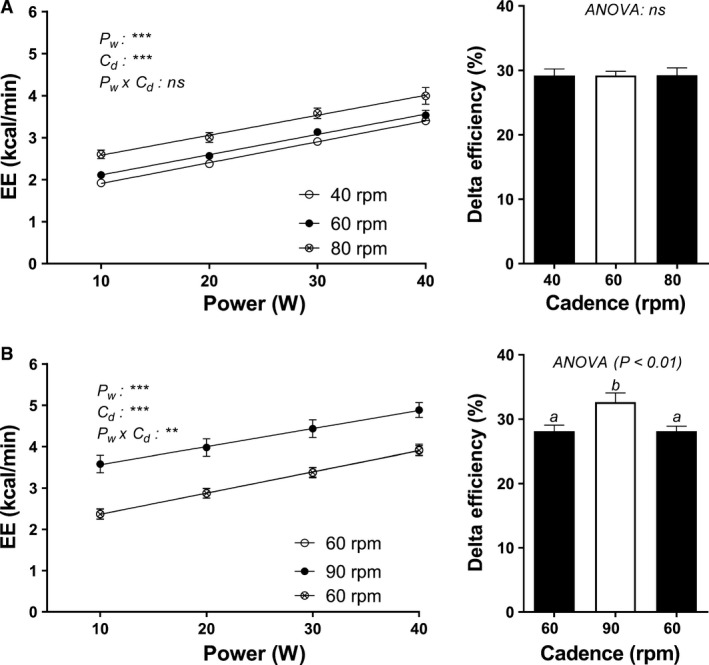
Energy expenditure (EE) as a function of power at different cadences (40, 60, 80 rpm, *panel A*; and 60, 90, 60 rpm, *panel B*). ANOVA indicates significant effect of power (P_w_), and cadence (C_d_); *panel A*). A significant effect of power (P_w_), cadence (C_d_), and interaction effect (P_w_ x C_d_) (*panel B*) was also shown. Delta efficiency (DE) values are presented in the bar charts on the right. Values are presented as Mean ± SEM. Values with different superscripts (*a, b*) are significantly different from each other (Tukey's HSD,* P* < 0.05).

The results of varying cadence according to protocol I (40, 60, 80 rpm) on EE and DE are presented in Figure**** 4 (panel A). The EE values are higher with increasing cadence across power, with higher values at 80 rpm in comparison to 40 and 60 rpm (ANOVA: *P* < 0.001), independently of gender. Calculations of DE values indicate no significant differences in DE across cadence of 40, 60 and 80 rpm, the DE values being 29.7, 29.8, and 29.3%, respectively. In contrast, the results of protocol II (60, 90, 60 rpm; Figure 4, panel B) show that when pedaling at 90 rpm, the slope of the EE‐power regression line is significantly lower when compared to pedaling at 60 rpm, thereby resulting in a significantly higher DE at 90 than at 60 rpm (32.6% vs. 28%, *P* < 0.01); this difference in cadence was observed both in men and women. When asked about cadence preference, the vast majority of subjects, independently of gender, reported preference for pedaling at 60 rpm rather than at 40, 80 or 90 rpm.

### Experiment III: repeatability & habituation

**Figure 5:**
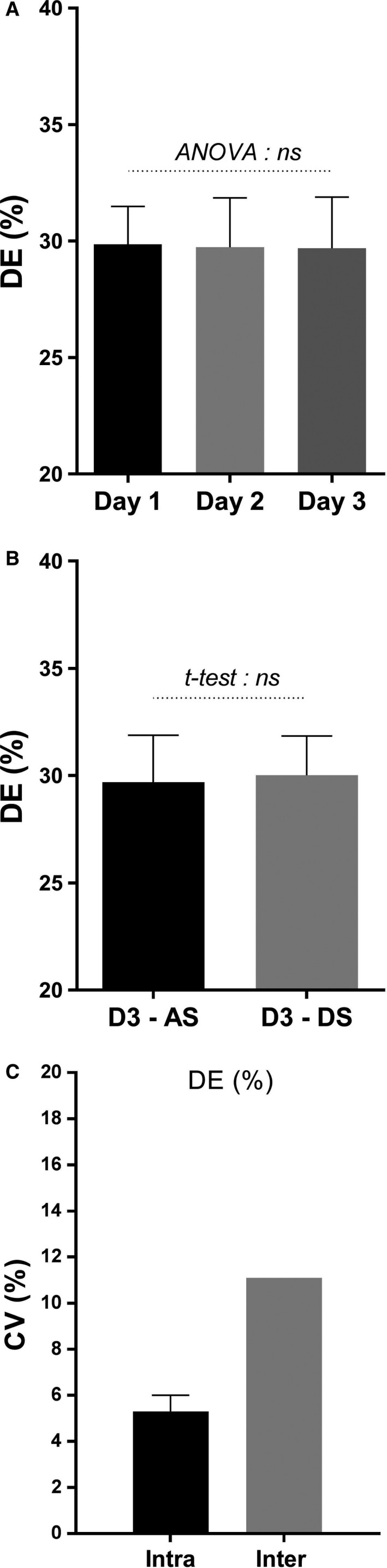
Repeatability of delta efficiency (DE) across three different days (D1, D2, D3). *Panel A*: DE values on different days (Mean ± SEM). *Panel B*: Ascending *(* AS) versus descending (DS) graded cycling DE. *Panel C*: Intra‐ and Inter‐ coefficient of variation (CV%) of DE. All values for DE (%) assessed by linear regression between 10 and 40W.

The results of this experiment performed on six subjects to investigate repeatability of DE on three different days are presented in Figure 5. Comparison of DE values during ascending power phase across the 3 days (D1 vs. D2 vs. D3; panel A) or on day 3 between ascending vs. descending power phase (D3‐AS vs. D3‐DS; panel B) show no systematic habituation or learning effect on DE, nor any influence of increasing vs. decreasing work rate. The result of this repeatability study (conducted over three different days) are presented in Table**** 1 as intra‐individual coefficient of variation (intra‐CV%) for cycling exercise efficiency, with the latter expressed in different ways. Compared to DE assessed by linear regression (CV = 5.2%), the CV for efficiencies calculated by the method of difference (DE‐md) or as net efficiency (NE) are 2‐3 folds higher for power outputs between 10 and 30 W and much larger for work efficiency (WE) at 10 W, while CV for gross efficiency (GE) was close or slightly lower than DE, varying between 3 and 4.5% depending upon the specific power output.

**Table 1:** Intra‐individual coefficient of variation (intra‐CV) for different expressions of efficiency during cycling

	Power (W)							∆ Power
	10		20		30		40	10–40
**DE**								**5.2**
SD								1.8
95% CI								3.4–7.1
**DE‐md**		**17.4**		**15.4**		**17.9**		**5.8**
SD		11.0		8.6		11.7		1.7
95% CI		5.8–28.9		6.3–24.4		5.6–30.1		4–7.6
**GE**	**4.5**		**3.2**		**4.4**		**2.9**	
SD	2.9		3.6		3.9		2.9	
95% CI	1.5–7.5		−0.6–7.1		0.3–8.4		−0.1–5.9	
**NE**	**12.8**		**9.2**		**9.6**		**5.4**	
SD	7.8		5.0		5.2		2.8	
95% CI	4.6–21		4–14.5		4.1–15.1		2.4–8.4	
**WE**	**42.6**		**16.5**		**10.6**		**3.9**	
SD	15.0		7.1		5.2		2.0	
95% CI	26.8–58.3		9–24		5.2–16.1		1.7–6	

Values in bold are mean; SD, standard deviation; CI, 95% confidence interval; DE, delta efficiency by the slope of the regression; DE‐md, delta efficiency by the method of difference; GE, gross efficiency; NE, net efficiency; WE, work efficiency**.**

### Experiment IV: potential determinants of inter‐individual variability in DE

The inter‐ and intra‐individual CVs for DE are presented in Figure 5 **,** panel C. DE shows modest inter‐individual variability for DE, with a CV of about 11% (*n* = 55), which is nonetheless twofold greater that the intra‐individual variability, the latter's CV being 5.2%. No significant correlation was found between DE and body weight, height or body composition (total fat mass, trunk (abdominal) fat, fat‐free‐mass, skeletal muscle mass, leg lean mass) in the whole population sample nor within each gender (Table 2). Furthermore, comparison of men (*n* = 26) and women (*n* = 29) from this entire cohort indicate no gender differences in DE, with mean ± SEM values of DE for men and women being 29.1 ± 0.6% and 28.6 ± 0.6% in men and women, respectively.

**Table 2:** Relationships between delta efficiency of low‐power cycling exercise and various anthropometric and body composition measures in inactive subjects

	All *(n = 55)*		Men *(n = 26)*		Women *(n = 29)*	
	*r*	*P*	*r*	*P*	*r*	*P*
Anthropometry						
Age (year)	−0.099	0.48	0.044	0.83	−0.280	0.16
Height (m)	0.089	0.53	0.156	0.46	−0.181	0.36
Weight (kg)	0.031	0.83	−0.227	0.27	0.143	0.48
BMI (kg/m^2^)	−0.007	0.96	−0.332	0.11	0.221	0.27
Body composition						
Fat‐free mass (Kg)	0.083	0.56	‐0.185	0.37	0.165	0.41
Skeletal muscle mass (kg)	0.091	0.52	−0.185	0.37	0.206	0.30
Leg lean mass (kg)	0.111	0.43	−0.013	0.95	0.067	0.74
Fat mass (kg)	0.095	0.50	0.116	0.58	0.063	0.76
Fat mass (%)	−0.061	0.67	−0.051	0.81	0.045	0.82
Trunk fat (%)	0.029	0.84	0.110	0.60	0.043	0.83

*P* refers to *P*‐value of the correlation coefficient (r); BMI, Body Mass Index.

## Discussion

The primary objective of this study was to assess, in inactive subjects, the reliability of low‐power cycling as an approach to study human variability in dynamic work efficiency across power outputs that are energetically comparable to everyday life activities (usually 1.5–4 METs) (Levine et al. 2000, 2001; Goh et al. 2016). It is shown here that in both men and women pedaling at 60 rpm, the EE‐power relationship during graded exercises at 5 min per workload in the low power range of 10–50W is robustly linear; its slope (and hence DE) has a high intra‐individual reproducibility. Furthermore, this cycling test, a non‐weight‐bearing activity, lasting less than 30 min, was easily performed and perceived as “light” by the untrained sedentary subjects.

### Exercise test perception

Indeed, in all individuals (men and women), the intensity of the cycling exercise was well‐tolerated. The levels of perceived exertion ‐ while pedaling at 60 rpm ‐ did not exceed the value of 12 (out of 20) on the Borg scale, and were hence in the “light” zone, including when cycling at the highest power output (50 W). The latter power output, however, seems to be the upper limit of perceived “light” exercise in the sedentary women. Furthermore, across 20–50 W, the perceived exertion ratings tended to be higher in women than men, and this was reflected in a significantly higher heart rate values in women than in men (by 8–17 bpm), particularly when cycling at power >20 W. It should be noted that in assessing the reliability of this low‐intensity exercise test, we did not perform the graded cycling exercise beyond the low power of 50W, and hence did not assess peak *V*O_2_ and associated variables of cardiopulmonary fitness. However, both men and women found the exercise test to be “very light” or “light” – an important criteria for feasibility and compliance pertaining to an exercise test to be applied to an inactive population. Furthermore, the vast majority of subjects, independently of gender, preferred to pedal at a cadence of 60 rpm despite the fact that EE increased with increasing cadence across the range of 40–90 rpm and DE was higher when pedaling at 90 rpm than at 60 rpm. Our findings of elevated EE at higher cadence across the low power range of cycling is in line with previous reports of higher EE with increasing cadence, despite a constant workload, during both low‐intensity and high‐intensity cycling (Takaishi et al. 1996; Rowland and Lisowski 2001; Hirano et al. 2015). In particular, in their investigation in sedentary (inactive) healthy young men performing low intensity (~60 W) cycling exercise, Hirano et al. (2015) found that EE was lower when pedaling at 35 rpm than at 75 rpm, and was associated with higher pedal force, lower peripheral oxygenation, and a lower central (ventilation rate, heart rate) response. Despite all these differences in physiological responses to variations in cadence, no differences were observed in Borg's rating of perceived exertion.

### EE‐power linearity

In terms of the energy cost of work performed relative to rest (i.e., METs), the increases in EE measured across 10–50 W varied in the range of 1.7–3.2 METs in men and 1.8–3.8 METs in women, which energetically corresponds to the low level physical activities of everyday life; these include household activities (cleaning, cooking, and bed making) typically in the range of 1.5–2 METS (Goh et al. 2016), low‐level spontaneous physical activity (fidgeting and pacing) ranging between 1.5 and 2.5 METs (Levine et al. 2000, 2001), and walking on a flat surface at speed in the range of 2–4 km per hour and ranging between 2 and 4 METs (Levine et al. 2000, 2001). Interestingly, the measured EE during unloaded (i.e., No‐load) cycling was found to be higher than that predicted for 0 W by the linear EE‐power relationship across 10–50 W. This discrepancy may be due to the extra EE induced by leg movement, work on the pedals and mechanical friction, as well as the cost of postural stabilization while pedaling, namely hand‐grip and trunk stabilization. Another reason might be that No‐load cycling involves pedaling against a power load that is slightly higher than 0 W or that the relationship between EE and power at the lowest end of power output (0–10 W) may be curvilinear rather than linear. Whatever the reasons for the higher EE values than predicted in 0–10 W range, the linearity of the EE‐power relationship across 10–50 W was very strong in both men and women. Indeed, for each of the 15 individuals in the linearity validation experiment, the *r* ^2^ value ‐ based upon five data points across 10–50 W exceeded 0.98 and was most often better than 0.99, thereby suggesting that the regression lines were an accurate representation of the EE‐power relationship in this range of low‐power cycling.

### Repeatability & habituation

The repeatability of the slope of the EE‐Power relationship (and hence DE), assessed on three separate occasions and on three different days was found to be good given an intra‐individual CV of 5.2% on average, and ranging between 3.4 and 7.1%. To the best of our knowledge, this study is the first to report intra‐individual variability in DE of low‐power cycling. This is shown here to be equally low in men as in women, only slightly higher than CV values often reported for basal metabolic rate (3–5%) (Henry 2005), and below the CV of 12% that we recently reported for the energy cost of intermittent, low‐level, isometric leg press (Sarafian et al. 2013). There were also no systematic habituation effect on DE since (1) the value of DE assessed under conditions of the exercise test with increasing power (ascending phase) was not different from that assessed during the exercise test with decreasing power (descending phase), and (2) there was no systematic trend across repetitions of the cycling test performed on three separate mornings with 2–3 days interval. In other words, there was no learning effect across days, and familiarization with the exercise test protocol does not seem necessary, which is in line with the findings that there is no effect of cycling experience on leg cycle ergometer efficiency for moderate‐to‐high intensity cycling (Nickleberry and Brooks 1996).

### Between‐study and within‐study inter‐individual variability

Interestingly, in the only reported reliability study during cycling exercise in active cyclists, Moseley and Jeukendrup (2001) found that in trained subjects pedaling at 80 rpm across moderate intensity to exhaustion (60 W to > 300 W), DE was about 26% on average, the within‐subject CV was about 6% and between‐subject variability about 12%. These values are similar to those found here in our untrained and inactive subjects pedaling in the low power output range of 10–50 W at 60 rpm. In contrast, we are surprised by the extent to which the values of DE in our inactive subjects (~28%) differed with those reported by Reger et al. (2013) where the average DE value was reported to be as high as 57% in the low power range of 10–40 W in active cyclists. Although we found here that DE was higher when pedaling between 10 and 40 W at 90 rpm than at 60 rpm, the increase in DE, although significant, resulted in an average value of DE of about 35% at 90 rpm, which is still much lower than the average value of DE reported by Reger et al. (2013) for similar low‐power cycling at 90 rpm. The latter authors also reported abnormally high DE values of 41% on average when their active cyclists performed moderate power cycling in the range of 50–120 W. These DE values contrast sharply with values reported for active cyclists (Suzuki 1979; Chavarren and Calbet 1999; Marsh et al. 2000; Moseley and Jeukendrup 2001; McDaniel et al. 2002; Moseley et al. 2004), namely DE in the range of 22.1–26%. Furthermore, the range of individual DE values in our study (23‐35%) is narrower compared to that reported by Reger et al. (2013), who found a twofold range of DE during low‐power cycling (36–78%). It should be noted that the cycling exercise tests in Reger's study were performed at non‐defined hours after a meal and by active cyclists pedaling at the high cadence of 90 rpm. Consequently, the considerably lower inter‐individual variability in our study than that in Reger et al. (2013) (CV of 11% vs. 24%, respectively) could partly reside in the more stringently standardized conditions of our study. Indeed, all our subjects were studied in the post‐absorptive state (10–12 h after an overnight fast), all women tested in the follicular phase of their menstrual cycle, all graded exercise tests performed in the morning after at least 30 min rest in a comfortable seat, and the use of a lower cadence (namely 60 rpm) which our inactive subjects considered to be most comfortable for leg cycling.

### Cycling exercise efficiency: arguments for delta efficiency

What constitutes the best expression of exercise efficiency has long been a subject for debate (Gaesser and Brooks 1975; Ettema and Loras 2009). For many researchers (in particular nutritionists), the GE, defined as the percentage of total energy expended (including resting EE) that produces external work, is considered to be the most relevant expression of efficiency. However, as Gaesser and Brooks (1975) have suggested, GE is a poor measure of the efficiency of muscular work as it distorts the linear relationship between EE and work rate such that efficiency appears to increase with increasing work rate. Indeed, within the low power output range of our study (Fig. 3), GE is found to increase in a curvilinear fashion. These apparent changes in efficiency are observed because the proportion of EE that is used to maintain basal functions (i.e., basal or resting EE) becomes smaller as total EE increases (Gaesser and Brooks 1975; Moseley and Jeukendrup 2001; Ettema and Loras 2009).

Consequently, as an alternative approach, a baseline resting EE can be subtracted from total EE in the calculation of efficiency. This is performed in the calculation of either net efficiency (where the baseline is taken as the EE at rest) or work efficiency (where the baseline is taken as the energy cost of unloaded or no‐load (0 W) cycling). However, both of these “baseline subtraction” methods of expressing efficiency are based upon the assumption that the baseline EE (at rest or during no‐load cycling) remains constant during exercise‐induced changes in EE (Moseley and Jeukendrup 2001). This assumption is not tenable because, as Moseley and Jeukendrup (2001) have argued, an increase in exercise intensity will result in changes in gastrointestinal blood flow, splanchnic processes, cardiac output, and ventilation rates. Such changes will inevitably result in an increase in the energy cost for achieving homeostasis during exercise, thereby altering the assumed baseline value (Moseley and Jeukendrup 2001).

In contrast, the expression of efficiency as delta efficiency is independent of the baseline value as it is assessed either as (1) the change in work performed between two trials, divided by the change in EE between these two trials (DE‐md), or (2) as the reciprocal of the slope (i.e., 1/slope) of the linear relationship of EE as a function of power output (i.e., DE) ‐ which is mathematically similar to using the intercept as a baseline subtraction. In line with other authors (Gaesser and Brooks 1975; Coyle et al. 1992; Moseley et al. 2004; Reger et al. 2013), we believe that DE, assessed by the method of linear regression across several power outputs), provides the most valid estimate of muscular efficiency as it expresses the incremental changes in EE relative to the incremental changes in actual work performed using the least square method. In other words, the slope reflects the energy cost of biological processes that increase with increasing power output, such as the increased energy needs for cardio‐respiratory and contracting skeletal muscles, whereas the intercept reflects the energy cost of biological processes at zero work rate that remain a constant.

In our analysis of the EE‐power relationship in the low power output range, we also found the individual regression lines to be an accurate representation of the EE‐power relationship (*r* ^2^ values very close to 1). Furthermore, although the repeatability of DE was lower than that of GE (CV of 5.2 vs. 3–4.5%), this difference during low‐power cycling is similar to that reported by Moseley et al. (2004) for moderate‐to‐high cycling exercise in trained cyclists where the CV of DE and GE were found to be 6.7% and 4.2%, respectively. DE assessed by linear regression is also more reliable than either NE or indeed DE‐md (which is calculated by method of difference between two measurements), where the within‐subject variability was much larger. Work efficiency (WE) values were extremely variable at very low power outputs, which is in line with findings of Hintzy‐Cloutier et al. (2003) and Reger et al. (2013). This is mostly because the power output level is so low that the relative inherent error of the measurement constitutes a large proportion of the given work load. Furthermore, of all the various forms of expression of efficiency, only DE is close to the theoretical value for muscle efficiency of 25–30% (Gaesser and Brooks 1975; Coyle et al. 1992).

Thus, from a consideration of both exercise physiology and statistical reliability, DE assessed by linear regression seems to be the most accurate expression of cycling exercise efficiency for metabolic phenotyping. Furthermore, the assumption that the DE of cycling in the seated position is independent of body weight is validated here for low‐power cycling across 10–50 W since our study indicates that inter‐individual variability in DE is not explained by variability in body weight in our population sample.

### Implications

In the evaluation of whether variability in exercise efficiency plays a role in predisposition to obesity, whether it is altered by the obese state or whether it contributes to adaptive thermogenesis operating to regulate body weight in response to weight loss or weight gain, it is important to appreciate the significance and limitations of its various forms of expression and also to know the reproducibility of its measurement. Indeed, in the absence of knowledge about the reliability of the measure of efficiency, it is difficult to interpret the results, as exemplified by contradictory findings regarding changes in cycling efficiency after weight loss. For example, the reports by Rosenbaum et al. (2003) and Goldsmith et al. (2010) that the gross or net efficiency of cycling at low power outputs (10, 25, and 50 W) in obese and lean subjects is increased after diet‐induced weight loss, and hence in a direction towards facilitating weight regain is in contradiction to the findings by Poole and Henson (1988) that the efficiency of cycling at 30–105 W (assessed as DE) was not altered by weight loss. These studies are difficult to compare and interpret, not only because of differences in the calculations of efficiency used across studies which could influence the interpretations of the findings and lead to different conclusions between studies, but also because the conflicting results could be due to poor reliability of the measure. Thus, in order to be able to understand the factors that determine or modulate efficiency, it is first necessary to establish what change or difference in efficiency can be reliably detected. On the basis of our studies here showing DE of 29% on average, an intra‐individual CV of 5%, and hence a standard deviation of 1.5% for within‐subject variability, power analysis with type‐I error (*α*) of 0.05 and a desired power (1−*β*) of 0.90 indicates that the sample size required to detect a 2 unit change in DE in a given population sample would be seven subjects. However, more subjects would be required for detecting a similar difference in DE between two population samples. Indeed, based upon our findings of an inter‐individual CV of 11% in DE (and hence a standard deviation of 3% for between‐subject variability), the sample size required to show a between‐group difference of 2 or 3 units in DE would be 35 or 16 subjects, respectively. Thus, while a sample size of 5–10 subjects may have sufficient power to detect a change in DE of 2–3 units in response to a given challenge (e.g., dieting, overfeeding), such a sample size would clearly be inadequate to detect between‐group differences in DE of 2–3 units (e.g., obese vs. lean, older vs. younger, active vs. inactive).

## Conclusions

In conclusion, the assessment of the efficiency of low‐power cycling analyzed by linear regression – and conducted within the range of the increase in EE for low‐intensity movements of everyday life (1.5–4 METs) – extends the capacity for metabolic phenotyping in the population. This low‐intensity cycling exercise test validated here has a low intra‐individual variability, and is independent of anthropometry. It is simple, easy to conduct and lasts <30 min. Even elderly and very obese individuals could perform such a low level of activity, as cycling in the seated position is a non‐weight‐bearing activity and well‐tolerated by most individuals. There is obviously no single standardized exercise test that can reflect the multitude of “real life” low level physical activities. In addition to non‐weight bearing exercise tests involving (1) dynamic (low‐power cycling) exercise reported here and (2) a standardized test of intermittent isometric (leg press) exercise reported previously (Sarafian et al. 2013), additional standardized test to reflect “weight bearing activities” are clearly warranted. In the meantime, the low‐power cycling exercise test described here opens up new avenues for research in human EE phenotyping, with implications for the role of altered efficiency of performing low‐level dynamic work in metabolic predisposition to leanness and fatness, as well as to assess the effect of obesity, aging and other diseased state on this aspect of movement efficiency. It could also be applied towards investigating potential changes in efficiency during the life cycle in relation to the postulated thrifty metabolism in those exposed to developmental programming (fetal/neonatal exposure in those born small), as a component of adaptive thermogenesis in response to weight loss/gain/regain, and during potential adaptations in energy metabolism that may occur during pregnancy and lactation.

## Conflict of Interest

The authors declare no conflict of interest.
